# Neuropsychopathological comorbidities in learning disorders

**DOI:** 10.1186/1471-2377-13-198

**Published:** 2013-12-13

**Authors:** Lucia Margari, Maura Buttiglione, Francesco Craig, Arcangelo Cristella, Concetta de Giambattista, Emilia Matera, Francesca Operto, Marta Simone

**Affiliations:** 1 Child Neuropsychiatry Unit, Department of Basic Medical Sciences, Neurosciences and Sense Organs of the “Aldo Moro” University of Bari, Piazza Giulio Cesare 1, Bari, Italy

**Keywords:** Learning disorders, Comorbidity, Language disorder, Motor coordination disorder, ADHD, Mood and anxiety disorders

## Abstract

**Background:**

Learning Disorders (LD) are complex diseases that affect about 2-10% of the school-age population. We performed neuropsychological and psychopathological evaluation, in order to investigate comorbidity in children with LD.

**Methods:**

Our sample consisted of 448 patients from 7 to 16 years of age with a diagnosis of LD, divided in two subgroups: Specific Learning Disorders (SLD), including reading, writing, mathematics disorders, and Learning Disorders Not Otherwise Specified (LD NOS).

**Results:**

Comorbidity with neuropsychopathologies was found in 62.2% of the total sample. In the LSD subgroup, ADHD was present in 33%, Anxiety Disorder in 28.8%, Developmental Coordination Disorder in 17.8%, Language Disorder in 11% and Mood Disorder in 9.4% of patients. In LD NOS subgroup, Language Disorder was present in 28.6%, Developmental Coordination Disorder in 27.5%, ADHD in 25.4%, Anxiety Disorder in 16.4%, Mood Disorder in 2.1% of patients. A statistically significant presence was respectively found for Language and Developmental Coordination Disorder comorbidity in LD NOS and for ADHD, mood and anxiety disorder comorbidity in SLD subgroup.

**Conclusions:**

The different findings emerging in this study suggested to promote further investigations to better define the difference between SLD and LD NOS, in order to improve specific interventions to reduce the long range consequences.

## Background

Learning Disorders (LD) affect about 2-10% of the school-age population. They are characterized by an academic functioning that is below the level that would be expected given their age, Intelligent Quotient (IQ) and grade level in school, and interfere significantly with academic performances or daily life activities that require reading, writing or calculation skills. LD are distinguished in Specific Learning Disorders (SLD) and Learning Disorders Non-Otherwise Specified (LD NOS), two categories separated for the clinic and care. SLD include Reading, Written Expression and Calculation Disorder. LD NOS refer to a disability in acquiring new knowledge and skills, that are not limited to one or more specific school areas (reading, writing, mathematics) but also extended to other areas.

LD are neurobiological disorders that are not diagnosed before school age, accompanying the subject during the course of their life. Genetic and acquired factors may occur alone or in combination in determining LD. Dyslexia is present in 35-45% of ascending and collaterals; the concordance is 84% in monozygotic and 50% in dizygotic twins; moreover it has been demonstrated a genetic association with different chromosomes, including 6, 15, 18 [1-3], and about 15 genes have been found associated with dyslexia [4,5]. Recently, Giraud and Ramus [6] reviewed the current literature and described a putative mechanistic model that linked neuronal micro-architecture of the auditory cortex to specific alterations of phonological processing. The authors suggested that dyslexia could be related to a disconnection syndrome, signaling candidate genes (DCDC2, KIAA0319, DYX1C1) associated with neuroanatomical alterations, involving both the white and the gray matter of a fronto-temporo-parietal network, suggestive of dysfunction in cortical connectivity. Several acquired factors have been also involved such as: childbirth dystocias, neonatal asphyxia, neonatal icterus, cardiorespiratory arrest, status epilepticus, low birth weight and preterm birth [7-9], smoker mother during pregnancy [10], exposure to more than 2 general anesthesia within the fourth year of life [11,12], parental history of alcoholism or substance abuse [13] and prenatal exposure to cocaine [14]. School, family and social context are also interweave with neurobiological and contribute to determine the multifactorial nature of LD.

LD show variable clinical features and often associate to other disorders [15], that complicate the LD clinical presentation. The knowledge of these aspects is important from a preventive and therapeutic point of view. For these reasons, in this study we analyzed comorbidities in LD, considering separately the SLD and LD NOS subgroups, in order to deepen clinical and etiopathogenic knowledge of these disorders and improve their treatment.

## Methods

### Subjects

The sample consisted of 448 patients with a diagnosis of Learning Disorder, formulated by a child and adolescent neuropsychiatrist. The patients were divided in 2 subgroups: SLD (including Reading, Writing and Calculation Disorders) and LD NOS. All participants had Italian as their first language and they referred to the Child Neuropsychiatric Unit of the University of Bari “Aldo Moro”, Italy, during the period between October 2010 and December 2012. The study was approved by the local ethical committee “Azienda Ospedaliero-Universitaria Consorziale Policlinico di Bari”. All children were recruited in this study after obtaining written informed consent by their parents. In addition, patients aged from 8 to 16 years of age, gave their written informed consent.

All patients underwent anamnesis (familiar, physiological, pathological and academic), physical and neurological examination, routine laboratory tests and electroencephalogram.

### Assessment

In this study we performed a clinical assessment of LD and specific comorbidities. Diagnosis was formulated, according to the diagnostic criteria of the Diagnostic and Statistical Manual of Mental Disorders Fourth Edition-Text Revision (DSM IV TR) [16], and it was supported by diagnostic standardized tests for neuropsychological and psychopathological evaluation.

Neuropsychological evaluation assessed cognitive level, reading, writing and arithmetic skills, visual motor abilities and language.

The cognitive level was assessed with Italian version of Wechsler Intelligence Scale for Children, Third Edition (WISC-III) [17], we could not use WISC IV [18] because it was validated for Italian language only on February 2012; Leiter International Performances Scale Revised-Visualization and Reasoning battery (Leiter-R) [19] was administered, as an alternative to WISC-III, to subjects with verbal disorders. The cognitive level was classified according to DSM IV TR criteria as follows: normal intellectual functioning, IQ > 84; borderline intellectual functioning, IQ 71–84; mild intellectual impairment, IQ 50/55-70; moderate intellectual impairment, IQ 35/40-50/55; severe intellectual impairment, IQ 20/25-35/40.

Academic achievement was assessed with the following batteries of tests, validated for the Italian language: MT Group Reading Tests for Primary School [20]; MT Group Reading Tests for Middle School [21]; MT Group Advanced Reading and Mathematics Tests [22] for the first biennium of Secondary School; Battery for the Evaluation of Developmental Dyslexia and Dysorthography [23] for Primary and Middle school; Evaluation Tests of Calculation Ability for Primary School [24] and Evaluation Tests of Calculation Ability and Problem Solving for Middle School [25].

Visual motor abilities were evaluated by using the Visual Motor Integration Developmental test [26].

Language was evaluated with the following tests, validated for the Italian language: Test for the Evaluation of Language [27] and Evaluation Test for the Language Comprehension [28].

Psychopathological evaluation included the following tests, assessing behavioral, anxiety, mood and interpersonal problems: Child Behaviour Checklist (CBCL, Achenbach, 2001) [29]; Kiddies Schedule for Affective Disorders and Schizophrenia for School-Age Children - Present and Lifetime Version (Kaufman et al. 2004) [30]; Screen for Childhood Anxiety Related Disorders [31]; Children Depression Inventory [32]; Conner’s Parent Rating Scale - Revised-Long Version [33]; Swanson Nolan and Pelham-IV [34].

The tests assessing cognitive level, skills of reading, writing and arithmetic and the CBCL were administered to all patients. The other listed tests were administered case by case to support diagnosis of neurodevelopmental and psychopathological disorders.

### Statistical analysis

All demographic and clinical variables were subjected to statistical analysis. Descriptive analysis was carried out for the sociodemographic characteristics of the two samples. To compare age and gender between SLD and LD NOS group, we used respectively Student’s t tests and Chi-square independence (*x*2) test. In order to examine the difference of neuropsychopathological comorbidities in a sample of SLD children compared with LD NOS children, the Chi-square independence was used. Statistical significance was considered for p-values ≤ 0.05. We used the Software Statistical Package for Social Science version 20.

## Results

The sample included 448 Caucasian patients (319 males and 129 females) aged from 7 to 16 years (mean age 10.45, DS ± 2.57). The SLD subgroup included 240 patients (53.5%), mean age of 10.39 years, SD ± 2.45. The subgroup of LD NOS included 208 subjects (46.4%), mean age of 10.2 years, SD ± 2.67.

Normal intellectual functioning was present in 218 patients (48.6%), borderline intellective functioning in 68 (16.2%), mild intellectual impairment in 132 (31.4%) and moderate intellectual impairment in 30 (7.2%).

In total sample, comorbidity with one or more neuropsychopathologies was found in 279 patients (62.2%). In SLD subgroup, one or more neuropsychopathological comorbidity were present in 140 patients (58.3%): Attention Deficit Hyperactivity Disorder (ADHD) combined or isolated in 63 (33%), 55 males and 8 females, Anxiety Disorder in 55 (28.8%), 39 males and 16 females, Language Disorder in 21 (11%), 18 males and 3 females, Developmental Coordination Disorder in 34 (17.8%), 30 males and 4 females, Mood Disorder in 18 (9.4%), 14 males and 4 females.

In LD NOS subgroup, one or more neuropsychopathological comorbidity were present in 139 patients (66.8%): Language Disorder in 54 (28.6%), 36 males and 18 females, Developmental Coordination Disorder in 52 (27.5%), 34 males and 18 females, Attention Deficit Hyperactivity Disorder (ADHD) combined or isolated in 48 (25.4%), 41 males and 7 females, Anxiety Disorder in 31 (16.4%), 16 males and 15 females, Mood Disorder in 4 (2.1%), 2 males and 2 females.

Statistically significant differences between the group of SLD and LD NOS for the presence of comorbidities (p = 0.64) did not emerge.

Considering each comorbidity, in the LD NOS subgroup, language and coordination disorders were more frequent with a statistical significant difference (p = 0.00); in the SLD subgroup, ADHD, anxiety and mood disorders comorbidity were more frequent with a statistical significant difference (p = 0.00).

Neuropsychopathological comorbidities in both subgroups are summarized in Table 1.

**Table 1:** **Neuropsycopathological comorbidity in SLD**^**1 **^**and LD NOS**^**2 **^**subgroups**

	**SLD**	**LD Nos**	**p**
**Number**	240	208	-
**Gender**			
Male	184	135	-
Female	56	73	-
**Age (mean ± sd)**	10.3 ± 2.45	10.2 ± 2.67	-
**Total Comorbidity %**	58.3%	66.8%	0.64
ADHD	33%	25.4%	0.00*
Anxiety disorder	28.8%	16.4%	0.00*
Mood disorder	9.4%	2.1%	0.00*
Language disorder	11%	28.6%	0.00*
Motor coordination disorder	17.8%	27.5%	0.00*

^1^Specific Learning Disorders; ^2^Learning Disorders Not Otherwise Specified; *p < 0.005.

## Discussion and conclusions

The epidemiology of LD is highly variable according to the type of LD, the spoken language and the tools used for the diagnosis. International epidemiological studies report a prevalence of 4-17% for dyslexia, 2-8% for dysorthography and 1-5% for dyscalculia [35-39]. SLD are more frequent in males than females [37,40].

Different languages have different writing systems and variations in prevalence depend on factors like the spelling opacity of each language. The Italian language has a shallow orthographical system, for this reason, we would expect a lower prevalence of SLD in Italy, where, however, the prevalence range is very large, between 0.88% and 10% [41-46]. In Italy, SLD represent about 30% of the users of local Neuropsychiatry Services and about 50% of patients which undergo rehabilitation [47].

Comorbidity are very common in Neuropsychiatric diseases, including LD, during developmental age. Understanding comorbidity is important because the presence of an additional disorder may affect the expression and severity of the clinical picture, requiring specific treatments and interventions. Patients with comorbidity compared to those without comorbidity usually exhibit more severe neurocognitive impairment, negative academic experience and social outcomes and lower treatment response.

Dyslexia is the most extensively investigated learning disorder in the national and international studies regarding its features and also its comorbidity.

Language disorders may precede or be associated with dyslexia. International studies have estimated that 30-40% of children with Specific Language Disorder receive a diagnosis of reading disorder later on [48-50] and a percentage between 55% [51] and 77% [52] of dyslexics meets the diagnostic criteria for Specific Language Disorder. In Italy, comorbidity with Specific Language Disorder has been found from 15% to 20% of dyslexic children [53,54]. These data support the hypothesis that Language Disorder and Dyslexia may have common genetic or etiologic factors and may be different manifestations of the same cognitive impairment [55-58].

In addition, children with LD often present motor, sensory, perceptual abnormalities [59-63]. Huc Chabrolle et al. [15] in a review found that the impairment of motor development is a feature of nearly 50% of patients with dyslexia and that dyslexia is common among dyspraxic patients. Motor coordination disorder was reported in a percentage from 10.3% to 26% of dyslexics [53,54]. These data support the “cerebellar theory” of dyslexia [64] according to which, the cerebellum, that is responsible for motor control and automate overlearned tasks (i.e. reading), in LD may exert an insufficient motor control influencing articulation, phonological representation and ability to form appropriate connections between graphemes and phonemes.

Comorbidity with other disorders also is known in LD. It is reported that approximately 60% of patients with dyslexia also meet the criteria for at least one neuropsychiatric disorder [65,66]. Comorbidity with ADHD is present from 10% to 50% of LD children, while comorbidity with dyslexia is present from 25 to 40% of ADHD patients [65,67-69]. Comorbidity with anxiety and mood disorders has been reported in some studies but in others no difference was detected in the symptoms of anxiety and depressed mood among children with and without LD [53,67,70-75].

We did not find comparative data between SLD and LD NOS in literature.

In our sample we detected a comorbidity with neuropsychopathological disorders in both analyzed subgroups with some differences. A more significant presence of language and motor coordination disorders was found in the LD NOS compared to SLD subgroup. This could be linked to a higher degree of functional impairment in LD NOS patients, which presented, in the majority of cases, intellectual disability that might interfere with the normal evolution of neurolinguistic and motor development.

A meaningful presence of ADHD, anxiety and depressed mood was detected in the SLD subgroup. Some old and less replicated studies have suggested that reading disorder might be the primary deficit which causes secondary symptoms of ADHD [76-79]. Recent data have shown that there are common cognitive deficits between the two disorders [80] according to a possible similar genetic etiology, as demonstrated by families studies in twins [81,82]. Our results might support these latter theories, as demonstrated by the higher frequency of ADHD in SLD patients. With regards to anxiety and depressed mood, a bi-directional relationship between anxiety, depression and academic achievement has recently been hypotized [83]. Anxiety and depressed mood could negatively impact learning process, alternatively children with LD may develop anxiety and mood problems, because they often reported adverse academic experiences. In our study we can assume that the symptoms of anxiety and depression are more frequent in the SLD subgroup, due to the greater introspective capacities of these children which are more aware of their difficulties compared to LD NOS patients, most of which have cognitive impairments.

In May 2013, it was published the DSM-5 [84] that provides the diagnostic category of LSD as a single, overall diagnosis. New criteria give detailed specifiers for the areas of reading, mathematics and written expression and specifiers for grade of severity (mild, moderate, severe). The classification system DSM-5 does not provide diagnostic category LD unspecified/NOS but admit that SLD can co-occurs with neurodevelopmental (e.g. ADHD, communication disorders, developmental coordination disorder, autistic spectrum disorder) or other mental disorders (e.g. anxiety disorders, depressive and bipolar disorders). Further investigations, in according to new classification criteria, are need to better define comorbidities and LSD prognostic profiles to implement appropriate intervention strategies.

## Competing interests

We confirm that we have read the journal’s position on issues involved in ethical publication and affirm that this report is consistent with those guidelines.

None of the authors has any competing interest to disclose.

All co-authors have seen and approved the final version of the paper and accept responsibility for the data presented.

There is no financial or others conflict of interest that may be related to the authors.

## Authors’ contributions

LM conceived of the study and participated in final approval of the version to be published, MB conceived of the study and participated in its design and coordination and helped to draft the manuscript, FC participated in the design of the study and performed the statistical analysis, AC carried out substantial contributions to conception, design and acquisition of data, CdG carried out substantial contributions to conception, design and acquisition of data, EM participated in the sequence alignment and drafted the manuscript FO carried out contributions to interpretation of data, MS carried out contributions to conception and design and acquisition of data. All authors have seen and approved the final version of the paper and accept responsibility for the data presented.

## Pre-publication history

The pre-publication history for this paper can be accessed here:

http://www.biomedcentral.com/1471-2377/13/198/prepub
